# Supplementary material for: Coupling of the AQUATOX and EFDC Models for Ecological Impact Assessment of Chemical Spill Scenarios in the Jeonju River, Korea
Source: Biology (Basel). 2020 Oct 19;9(10):340. doi: 10.3390/biology9100340 (PMC7603242; doi:10.3390/biology9100340)
Supplement: Supplementary file 1 [file biology-09-00340-s001.pdf]

biology MDPI

Table S1. Water quality and atmospheric conditions in Jeonju River on 1 July, 2014 (Data according to website of the Water Environment Information System (WEIS; http://water.nier.go.kr/publicMain/mainContent.do) and https://www.google.co.kr/maps?hl=en&tab=wl1).

Parameter	Value	References
**Observing date**	2014.07.01	-
**pH**	7.5	WEIS
**Temperature (℃)**	26	WEIS
**Dissolved oxygen (mg/L)**	7	WEIS
**COD (mg/L)**	6	WEIS
**BOD (mg/L)**	2	WEIS
**Maximum depth (m)**	0.8	WEIS
**NO3 (mg/L)**	0.384	WEIS
**NH3 and NH4 (mg/L)**	0.182	WEIS
**Total Phosphorus (mg/L)**	0.074	WEIS
**Total Nitrogen (mg/L)**	1.241	WEIS
**Chl a (mg/m^3)**	12	WEIS
**PO4 (mg/L)**	0.054	WEIS
**Latitude**	35.9	Google maps
**Wind Velocity (m/s)**	1.39	WEIS
**Mean Light Intensity (Ly/d)**	307	WEIS
**Mean Evaporation Capacity**	46.8	WEIS

Table S2. Density of diatoms, ash-free dry mass (AFDM) and dry mass (DM) in Jeonju River.

Species Name	Density in Jeonju River (cells/cm^3)	AFDM of One Individual (ng C/cell)	AFDM Density of Species in the Site (mg C/L)	DM Density of Species in the Site (mg/L)
Cocconeis placentula	249	4.40×10^{-2}	1.10×10^{-2}	3.04×10^{-2}
Cyclotella meneghiniana	249	8.02×10^{-3}	2.00×10^{-3}	5.55×10^{-3}
Cymbella cistula	12724	6.10×10^{-3}	7.76×10^{-2}	2.16×10^{-1}
Fragilaria elliptica	1497	1.63×10^{-2}	2.43×10^{-2}	6.76×10^{-2}
Fragilaria ulna	1746	1.63×10^{-2}	2.84×10^{-2}	7.88×10^{-2}
Gomphonema lagenula	249	1.63×10^{-2}	4.05×10^{-3}	1.12×10^{-2}
Melosira varians	20958	1.29×10^{-2}	2.71×10^{-1}	7.53×10^{-1}
Navicula minima	4740	1.80×10^{-2}	8.51×10^{-2}	2.36×10^{-1}
Navicula pupula	499	5.68×10^{-3}	2.83×10^{-3}	7.87×10^{-3}
Navicula subminuscula	1497	1.80×10^{-2}	2.69×10^{-2}	7.47×10^{-2}
Nitzschia amphibia	7235	1.99×10^{-2}	1.44×10^{-1}	3.99×10^{-1}
Nitzschia inconspicua	249	1.99×10^{-2}	4.95×10^{-3}	1.37×10^{-2}
Nitzschia palea	2495	2.91×10^{-2}	7.26×10^{-2}	2.02×10^{-1}

Reimeria sinuata	249	1.63×10^{-2}	4.05×10^{-3}	1.12×10^{-2}

Table S3. Density of invertebrates and DM in Jeonju River.

Species Name	Density in Jeonju river (individuals/m²)	DM of individual species (g/individual)	DM density of species in the site(g/m²)	Length-Weight coefficient a	Length-Weight coefficient b	Reference for Length-Weight coefficient
Acentrella gnom	222	8.04×10^{-4}	1.79×10^{-1}	0.00962	2.75	[26]
Acentrella sibirica	19	8.04×10^{-4}	1.53×10^{-2}	0.00962	2.75	[26]
Antocha KUa	7	2.07×10^{-3}	1.45×10^{-2}	0.0305	2.62	[29]
Asellus hilgendorfii	74	2.97×10^{-3}	2.20×10^{-1}	0.0054	2.948	[23]
Baetiella tuberculata	252	4.25×10^{-3}	1.07×10^{-1}	0.00525	2.73	[30]
Baetis fuscatus	1066	6.72×10^{-3}	7.16×10^{-1}	0.00438	2.689	[25]
Caenis nishinoae	104	6.58×10^{-2}	6.84	4	2.02	[28]
Calopteryx japonica	4	3.22×10^{-1}	1.29	0.0078	2.792	[24]
Chaetogaster limnaei	15	0	0	0	0	No matched species
Cheumatopsyche brevilineata	1188	5.23×10^{-3}	6.21	0.00848	2.79	[30]
Chironomidae sp.	311	3.92×10^{-3}	1.22	0.001696	3.23	[30]
Euthraulus altioculus	4	2.56×10^{-3}	1.03×10^{-2}	0.0071	2.832	[24]
Dugesia japonica	440	0	0	0	0	No matched species
Ecdyonurus kibunensis	93	7.07×10^{-4}	6.57×10^{-2}	0.00664	2.9	[30]
Ecdyonurus levis	85	5.27×10^{-3}	4.48×10^{-1}	0.00664	2.9	[30]
Epeorus pellucidus	144	1.01×10^{-2}	1.45	0.00664	2.9	[30]
Ephemera orientalis	4	1.01×10^{-1}	4.04×10^{-1}	0.117	2.257	[27]
Serratella setigera	48	7.64×10^{-4}	3.67×10^{-3}	0.00103	2.676	[24]
Erpobdella lineata	52	0	0	0	0	No matched species
Hydropsyche kozhantschikovi	52	1.62×10^{-2}	8.43×10^{-1}	0.00848	2.79	[30]
Hydropsyche valvata	104	1.62×10^{-2}	1.69	0.00848	2.79	[30]

Hydroptila KUa	4	1.82×10^{-4}	7.27×10^{-4}	0.00848	2.79	[24]
Labiobaetis atrebatinus	26	2.81×10^{-3}	7.29×10^{-2}	0.00848	2.79	[30]
Limnodrilus gotoi	26	0	0	0	0	No matched species
Nigrobaetis bacillus	285	7.56×10^{-4}	2.15×10^{-1}	0.00848	2.79	[30]
Physa acuta	74	1.26×10^{-2}	9.35×10^{-1}	0.07	2.67	[30]
Mataeopsephus Kua	4	6.13×10^{-4}	2.45×10^{-3}	0.0077	2.91	[24]
Rhoenanthus coreanus	7	1.08×10^{-1}	7.58×10^{-1}	0.0071	2.832	[24]
Toryx tagoi	4	0	0	0	0	No matched species
Teloganopsis punctisetae	433	6.77×10^{-4}	$2.93x10^{-1}$	0.0071	2.832	[24]

Table S4. Density of fish and DM in Jeonju River.

Species Name	Density in BJeonjuCheon5 Point (individuals/12000m²)	WM of Individual Species (g/individual)	DM Density of Species in the Site (g/m²)	Wet to Dry Coefficient (g/g)	Reference for Length-Weight coefficient
Carassius auratus	4	2.35×10^{2}	1.57×10^{-2}	5	FishBase
Pseudogobio esocinus	45	3.30×10	2.48×10^{-2}	5	FishBase
Squalidus gracilis	15	6.09	2.06×10^{-3}	3.7	FishBase
Zacco platypus	47	3.01×10	3.19×10^{-2}	3.7	FishBase

Table S5. Acute toxicity values for the species in Jeonju River (some data according to the website https://cfpub.epa.gov/ecotox/search.cfm, while the other data calculated by the Interspecies Correlation Estimation (ICE) on https://www3.epa.gov/webice/iceDownloads.html).

Species Name (AQUATOX)	LC50 (μg/L)	Experiment time (h)	LC50 Reference	EC50 Growth (μg/L)	Experiment Time (hours)	EC50 Reference	Note (Toxicity Record in AQUATOX)
Golden Shiner	1.60×10^{4}	24	ECOTOX, adult	8.04×10^{3}	168	ECOTOX	Minnow
Minnow	1.60×10^{4}	24	ECOTOX, adult	8.04×10^{3}	168	ECOTOX	Minnow
Stoneroller	1.60×10^{4}	24	ECOTOX, adult	8.04×10^{3}	168	ECOTOX	Minnow
Mayfly(Baetis)	6.49×10^{4}	48	[35]	3.26×10^{4}	48	using Minnow LC50/EC50 ratio	Chironomid
Caddisfly	5.80×10^{4}	96	ECOTOX	2.9×10^{4}	96	using	Stonefly

						Minnow LC50/EC50 ratio	
Asian mud snail	5.56×10[4]	96	ECOTOX, physa snail		96	using Minnow LC50/EC50 ratio	Ostracod
Chironomid	6.49×10[4]	48	[35]	3.26×10[4]	48	using Minnow LC50/EC50 ratio	Chironomid
Odonata	5.80×10[4]	96	ECOTOX	2.9×10[4]	96	using Minnow LC50/EC50 ratio	Stonefly
Isopod	1.35×10[3]	24	ICE model Fathead minnow; 0.95 confidence interval is [358.4,5081]	6.78×10[2]	24	using Minnow LC50/EC50 ratio	Amphipod
Riffle beetle	1.35×10[3]	24	ICE model Fathead minnow; 0.95 confidence interval is [358.4,5081]	6.78×10[2]	24	using Minnow LC50/EC50 ratio	Amphipod

Table S6. Toxicity data of toluene for aquatic plants (data according to the website https://cfpub.epa.gov/ecotox/search.cfm).

Species Name (AQUATOX)	LC50 (μg/L)	Experiment time (hours)	LC50 Reference	EC50 Photosynthesis (μg/L)	Experiment time (h)	EC50 Reference	Note (Toxicity record in AQUATOX)
Melosira	1.51×10[4]	95	[36], algae	2.00×10[4]	3	ECOTOX, diatom	Diatoms
Navicula	1.51×10[4]	95	[36], algae	2.00×10[4]	3	ECOTOX, diatom	Peri, Cyclotell
Nitzchia	1.51×10[4]	95	[36], algae	2.00×10[4]	3	ECOTOX, diatom	Diatoms

Table S7. Species matching of local species to that in the library of AQUATOX (animals).

Species group	Species Name	Matched Species in AQUATOX (1)	Matched Species in AQUATOX (Final)
Fish	*Carassius auratus*	Golden Shiner	Golden Shiner

	Pseudogobio esocinus	Stoneroller	Stoneroller
	Squalidus gracilis	Minnow	Minnow
	Zacco platypus	Minnow	Minnow
Invertebrates	*Acentrella gnom*	Mayfly(Baetis)	Mayfly(Baetis)
	Acentrella sibirica	Mayfly(Baetis)	Mayfly(Baetis)
	Antocha KUa	Chironomid	Chironomid
	Asellus hilgendorfii	Isopod	Isopod
	Baetiella tuberculata	Mayfly(Baetis)	Mayfly(Baetis)
	Baetis fuscatus	Mayfly(Baetis)	Mayfly(Baetis)
	Caenis nishinoae	Tricorythodes	Mayfly(Baetis)
	Calopteryx japonica	Odonata	Odonata
	Chaetogaster limnaei	-	-
	Cheumatopsyche brevilineata	Caddisfly	Caddisfly
	Chironomidae sp.	Chironomid	Chironomid
	Euthraulus altioculus	Tricorythodes	Mayfly(Baetis)
	Dugesia japonica	-	-
	Ecdyonurus kibunensis	Mayfly(Baetis)	Mayfly(Baetis)
	Ecdyonurus levis	Mayfly(Baetis)	Mayfly(Baetis)
	Epeorus pellucidus	Mayfly(Baetis)	Mayfly(Baetis)
	Ephemera orientalis	Mayfly(Baetis)	Mayfly(Baetis)
	Serratella setigera	Mayfly(Baetis)	Mayfly(Baetis)
	Erpobdella lineata	-	-
	Hydropsyche kozhantschikovi	Caddisfly	Caddisfly
	Hydropsyche valvata	Caddisfly	Caddisfly
	Hydroptila KUa	Caddisfly	Caddisfly
	Labiobaetis atrebatinus	Mayfly(Baetis)	Mayfly(Baetis)
	Limnodrilus gotoi	-	-
	Nigrobaetis bacillus	Mayfly(Baetis)	Mayfly(Baetis)
	Physa acuta	Asian mud snail	Asian mud snail
	Mataeopsephus Kua	Riffle beetle	Riffle beetle
	Rhoenanthus coreanus	Mayfly(Baetis)	Mayfly(Baetis)
	Toryx tagoi	-	-
	Teloganopsis punctisetae	Mayfly(Baetis)	Mayfly(Baetis)

Table S8. Species matching of local species to that in the library of AQUATOX (diatoms) (Peri-Navicula and Peri-Nitzschia were also converted into Phyto-Navicula and Phyto-Nitzchia).

Species group	Species Name	Matched Species in AQUATOX (1)	Matched Species in AQUATOX (Final)
Diatoms	*Cocconeis placentula*	Periphyton,Cocconeis	Peri, Navicula
	Cyclotella meneghiniana	Cyclotella nana	Peri, Navicula
	Cymbella cistula	Periphyton,Cocconeis	Peri, Navicula
	Fragilaria elliptica	Peri, Fragilaria	Peri, Navicula
	Fragilaria ulna	Peri, Fragilaria	Peri, Navicula
	Gomphonema lagenula	Peri, Navicula	Peri, Navicula
	Melosira varians	Melosira varians (Additional species, Phyto and Peri)	Melosira varians (Additional species, Phyto and Peri)
	Navicula minima	Peri, Navicula	Peri, Navicula
	Navicula pupula	Peri, Navicula	Peri, Navicula
	Navicula subminuscula	Peri, Navicula	Peri, Navicula
	Nitzschia amphibia	Peri, Nitzschia	Peri, Nitzschia
	Nitzschia inconspicua	Peri, Nitzschia	Peri, Nitzschia

Table S9. Segmentation of the total stretch of the target site.

Segment	A	B (Accident Point)	C	D	E
Range	S1 to S2	S2 to S3	S3 to S4	S4 to S5	S5 to S6
Distance from S1	0–560 m	560–1.2 km	1.2–1.78 km	1.78–2.34 km	2.34–2.86 km
Surface area	41,745 m^2	47,902 m^2	55,253 m^2	54,513 m^2	30,245 m^2

Table S10. Boundary conditions of Jeonju River.

River	Observation Site	Flow Rate (m^3/s)			Reference	Date
		High Water Flow (95th)	Median Water Flow (185th)	Low Water Flow (275th)		
Mankyeong	Daecheon	15.032	10.444	7.002	WEIS	2017
Sam	Hyoja	16.032	10.444	7.002	WEIS	2017

Table S11. Toluene spill accident (perturbed) scenarios for the AQUATOX — environmental fluid dynamic source code (EFDC) model.

	Scenario 1	Scenario 2	Scenario 3	Scenario 4
Quantity of Total Spilled Toluene (kg)	3.0×10	3.0×10^2	3.0×10^3	3.0×10^4
Concentration of Inflow Toluene (μg/L)	8.7×10^8	8.7×10^8	8.7×10^8	8.7×10^8
Volueme of Inflow Loading (m^3/day)	3.4×10^{-2}	3.4×10^{-1}	3.4	3.4×10

Table S12. Food preference ratio table for the species in Jeonju River.

Species name	Mayfly (Baetis)	Riffle beetle	Caddisfly	Isopod	Chironomid	Asian mud snail	Odonata	Golden Shiner	Minnow	Stoneroller
**R detr sed**				7.8%	1.0%					
**L detr sed**	5.3%	5.3%		54.7%	99.0%	100.0%				23.3%
**R detr part**									4.1%	
**L detr part**			16.7%					7.9%	4.1%	
**Eunotia 002**	15.8%	31.6%		6.2%					9.2%	10.0%
**Peri, Navicula**	15.8%	31.6%		6.2%					9.2%	10.0%
**Peri, Nitzschia**	15.8%	31.6%		6.2%					9.2%	10.0%
**Eunotia 002_Phy**	15.8%		16.7%	6.2%					9.2%	
**Phyto, Nitzschia**	15.8%		16.7%	6.2%					9.2%	
**Phyto, Navicula**	15.8%		16.7%	6.2%					9.2%	
**Mayfly (Baetis)**							33.3%	23.8%	7.2%	
**Riffle beetle**								23.8%	7.2%	
**Caddisfly**								23.8%	7.2%	
**Isopod**							33.3%	10.3%	5.0%	
**Chironomid**			33.3%				33.3%	10.3%	5.0%	
**Asian mud snail**										
**Odonata**									4.6%	46.7%
**Golden Shiner**										
**Minnow**										
**Stoneroller**										

Table S13. Egestion fraction table for the species in Jeonju River.

Species name	Mayfly (Baetis)	Riffle beetle	Caddisfly	Isopod	Chironomid	Asian mud snail	Odonata	Golden Shiner	Minnow	Stoneroller
**R detr sed**				1.00	1.00					
**L detr sed**	0.50	0.53		0.60	0.50	0.16				0.50
**R detr part**									1.00	
**L detr part**			0.30					0.50	0.80	
**Eunotia 002**	0.30	0.16		0.30					0.20	0.30
**Peri, Navicula**	0.30	0.16		0.30					0.20	0.30
**Peri, Nitzschia**	0.30	0.16		0.30					0.20	0.30
**Eunotia 002_Phy**	0.30		0.16	0.30					0.20	
**Phyto, Nitzschia**	0.30		0.16	0.30					0.20	
**Phyto, Navicula**	0.30		0.16	0.30					0.20	
**Mayfly (Baetis)**							0.15	0.16	0.16	
**Riffle beetle**								0.16	0.16	
**Caddisfly**								0.16	0.16	
**Isopod**							0.15	0.16	0.16	
**Chironomid**			0.15				0.15	0.16	0.16	
**Asian mud snail**										
**Odonata**									0.16	0.16
**Golden Shiner**										
**Minnow**										
**Stoneroller**										

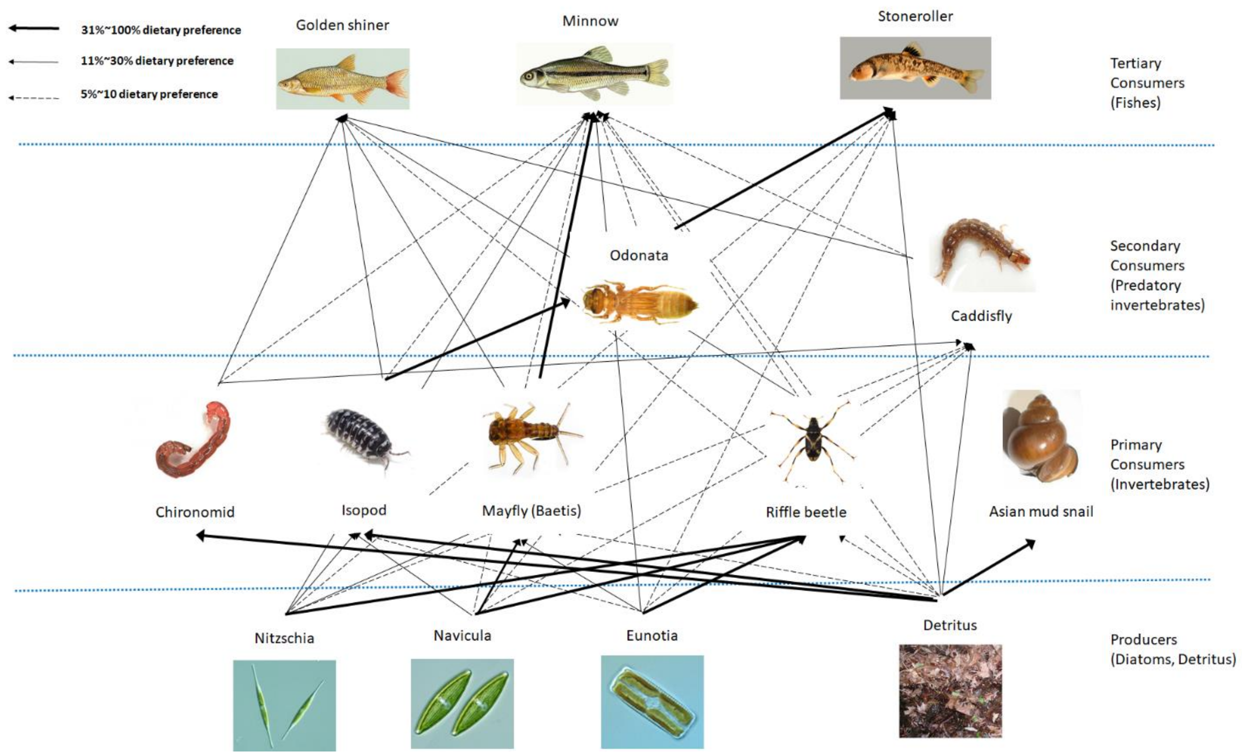

Figure S1. The aquatic species and food web in Jeonju River.

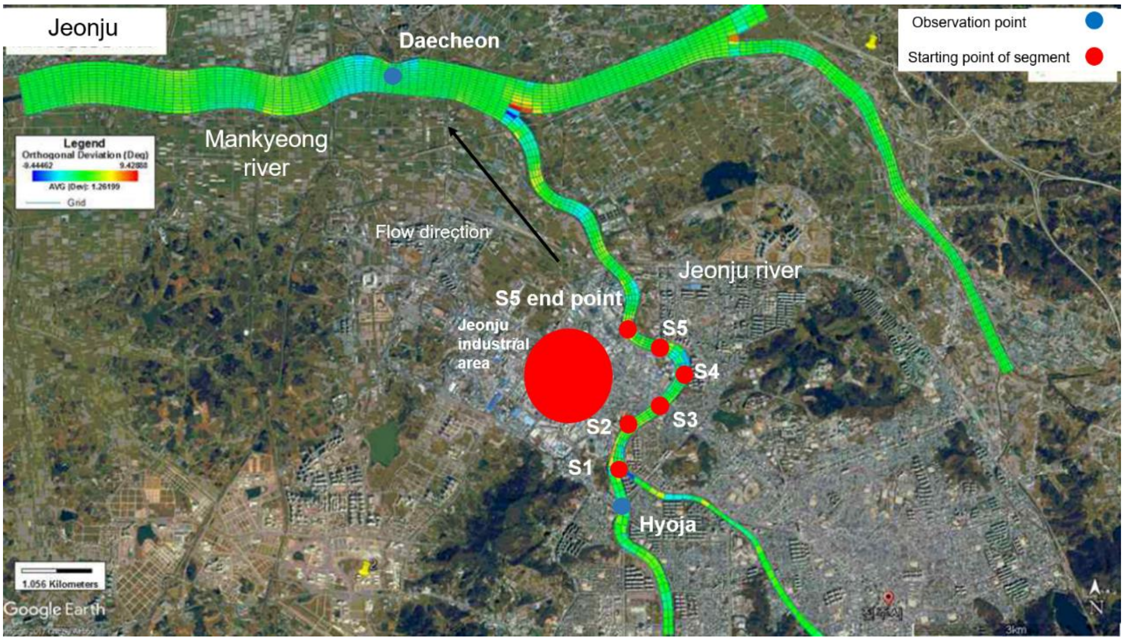

Figure S2. The EFDC map of Jeonju River.

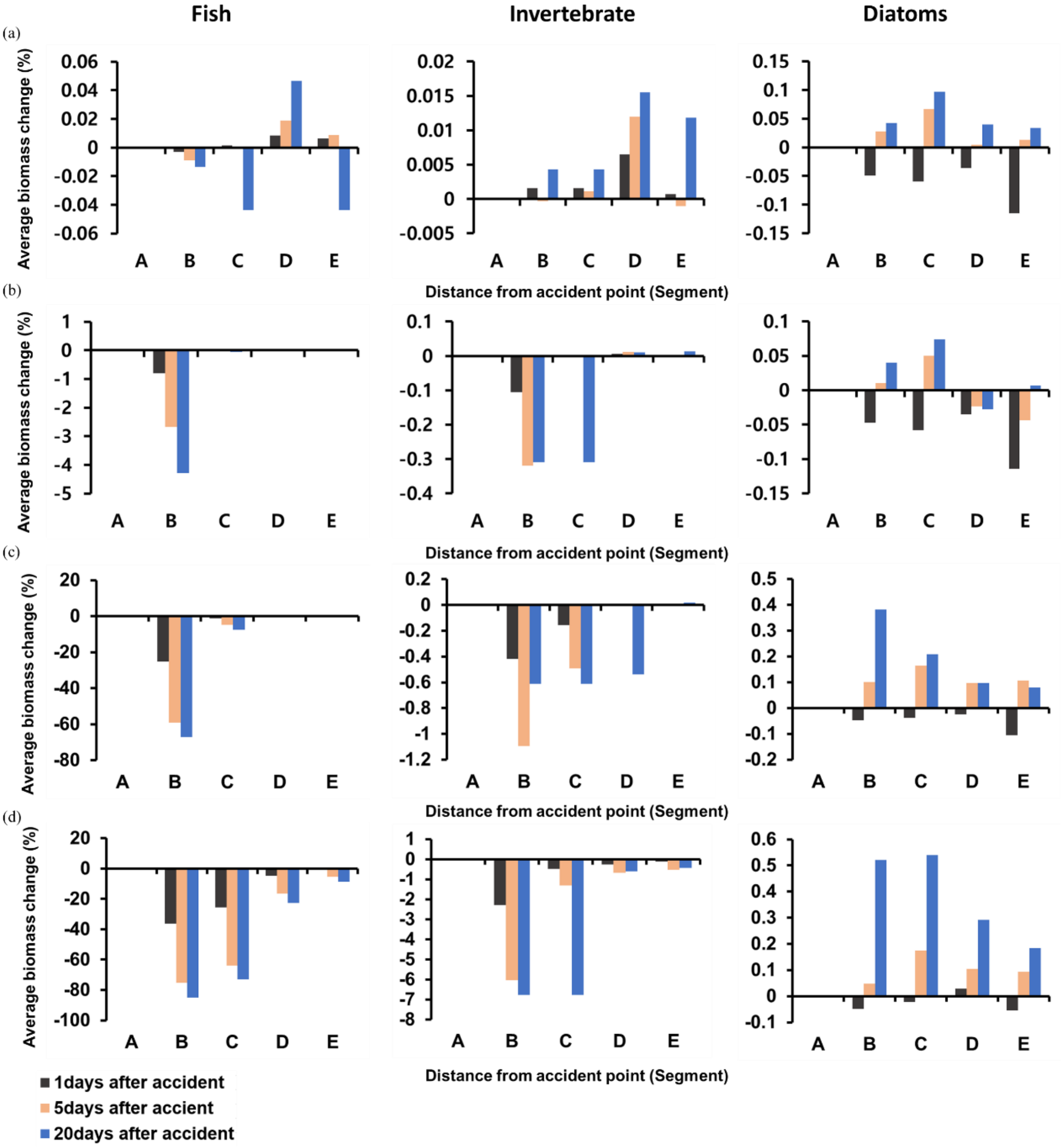

Figure S3. The relative variation of the average biomass of fish, invertebrates, and diatoms compared to the control (%) for 1–20 days after a (a) 30 kg, (b) 300 kg, (c) 3000 kg, and (d) 30,000 kg spill of toluene in Jeonju River.

Publisher's Note: MDPI stays neutral with regard to jurisdictional claims in published maps and institutional affiliations.

© 2020 by the authors. Licensee MDPI, Basel, Switzerland. This article is an open access article distributed under the terms and conditions of the Creative Commons Attribution (CC BY) license (http://creativecommons.org/licenses/by/4.0/).
